# Development and internal validation of prediction models for colorectal cancer survivors to estimate the 1-year risk of low health-related quality of life in multiple domains

**DOI:** 10.1186/s12911-020-1064-9

**Published:** 2020-03-12

**Authors:** Dóra Révész, Sander M. J. van Kuijk, Floortje Mols, Fränzel J. B. van Duijnhoven, Renate M. Winkels, Huub Hoofs, I Jmert Kant, Luc J. Smits, Stéphanie O. Breukink, Lonneke V. van de Poll-Franse, Ellen Kampman, Sandra Beijer, Matty P. Weijenberg, Martijn J. L. Bours

**Affiliations:** 1 Department of Epidemiology, GROW – School for Oncology and Developmental Biology, Maastricht University, P. Debyeplein 1, 6200 MD Maastricht, the Netherlands; 2 Department of Medical and Clinical Psychology, CoRPS – Center of Research on Psychology in Somatic diseases, Tilburg University, Warandelaan 2, 5037 AB Tilburg, the Netherlands; 3 Clinical Epidemiology and Medical Technology Assessment, Maastricht University Medical Centre+, P. Debyelaan 25, PO Box 5800, Maastricht, 6202 AZ the Netherlands; 4 Netherlands Comprehensive Cancer Organisation (IKNL), Godebaldkwartier 419, 3511 DT Utrecht, the Netherlands; 5 Division of Human Nutrition, Wageningen University & Research, Stippeneng 4, 6708 WE Wageningen, the Netherlands; 6 Department of Public Health Sciences, Penn State Cancer Institute, 500 University, Hershey, PA 17033 USA; 7 Department of Epidemiology, CAPHRI School for Public Health and Primary Care, Faculty of Health, Medicine and Life Sciences, Maastricht University, P. Debyeplein 1, 6200 MD Maastricht, the Netherlands; 8 Department of Surgery, Maastricht University Medical Centre, P. Debyelaan 25, 6229 HX Maastricht, the Netherlands; 9 Department of Psychosocial Oncology and Epidemiology, Netherlands Cancer Institute, Plesmanlaan 121, 1066 CX Amsterdam, the Netherlands

**Keywords:** Colorectal cancer, Cancer survivors, Quality of life, Prediction models, Model development, Internal validation

## Abstract

**Background:**

Many colorectal cancer (CRC) survivors experience persisting health problems post-treatment that compromise their health-related quality of life (HRQoL). Prediction models are useful tools for identifying survivors at risk of low HRQoL in the future and for taking preventive action. Therefore, we developed prediction models for CRC survivors to estimate the 1-year risk of low HRQoL in multiple domains.

**Methods:**

In 1458 CRC survivors, seven HRQoL domains (EORTC QLQ-C30: global QoL; cognitive, emotional, physical, role, social functioning; fatigue) were measured prospectively at study baseline and 1 year later. For each HRQoL domain, scores at 1-year follow-up were dichotomized into low versus normal/high. Separate multivariable logistic prediction models including biopsychosocial predictors measured at baseline were developed for the seven HRQoL domains, and internally validated using bootstrapping.

**Results:**

Average time since diagnosis was 5 years at study baseline. Prediction models included both non-modifiable predictors (age, sex, socio-economic status, time since diagnosis, tumor stage, chemotherapy, radiotherapy, stoma, micturition, chemotherapy-related, stoma-related and gastrointestinal complaints, comorbidities, social inhibition/negative affectivity, and working status) and modifiable predictors (body mass index, physical activity, smoking, meat consumption, anxiety/depression, pain, and baseline fatigue and HRQoL scores). Internally validated models showed good calibration and discrimination (AUCs: 0.83–0.93).

**Conclusions:**

The prediction models performed well for estimating 1-year risk of low HRQoL in seven domains. External validation is needed before models can be applied in practice.

## Background

The number of colorectal cancer (CRC) survivors is increasing as a result of rising incidence rates related to population ageing and a more widespread adoption of western lifestyles and of rising survival rates due to improved treatments and implementation of screening programs [1–3]. CRC survivors are often not only concerned about how *long* they will survive after treatment (quantity of life) but also how *well* they will survive (quality of life), because after diagnosis and treatment many survivors continue to experience physical and psychosocial problems and long-lasting and late treatment effects that can have a major impact on their health-related quality of life (HRQoL) [2, 4–6]. To anticipate the occurrence of potential HRQoL problems and enable appropriate preventive actions, it is important to identify individual survivors who have an increased risk of experiencing HRQoL problems in the future. Estimation of the future risk of low HRQoL in multiple domains, such as global quality of life and several functioning domains (e.g. physical, social and role functioning), can offer opportunities for tailoring of appropriate preventive interventions aimed at safeguarding the HRQoL of CRC survivors, for example through health behavioral interventions [7–13]. However, tools for risk estimation of future HRQoL are currently not available for CRC survivors.

In order to identify CRC survivors at risk of having low HRQoL in the future, accurate risk estimation must be based on relevant predictive factors incorporated in risk prediction models. Previous studies have investigated associations of clinical, personal, lifestyle, and psychosocial factors with HRQoL in CRC survivors [14–16]. Although such research enhances our understanding of the disease and treatments effects on HRQoL, it remains to be investigated whether these factors are useful for risk estimation. No study has yet incorporated these factors into risk prediction models, which are statistical models that enable estimation of the risk of some outcome variable based on a collection of predictors that should be interpreted in combination and not in isolation [17]. Several models have been developed to predict overall or progression-free survival after CRC, both using clinical and comorbidity factors, thereby aiding the decision-making process regarding treatment choices for individual CRC patients [18–21]. Up to date, however, no models have been developed for predicting future HRQoL in CRC survivors, whilst such prognostic models could be invaluable for identifying individuals at risk of future low HRQoL, preferably in multiple domains to estimate personal risk profiles that can indicate future problems in specific HRQoL domains [22–24].

Risk prediction models should be developed and rigorously tested according to a systematic research approach [25, 26]. Prediction research generally consists of three successive steps: 1. model development and internal validation, 2. external model validation, and 3. clinical impact evaluation. Development of a prediction model should always start with an evidence-based selection of candidate predictors potentially eligible for inclusion in an appropriate statistical model [17, 25, 26]. As starting point for developing a prediction model for HRQoL of CRC survivors, we have therefore provided a broad overview of candidate predictors of HRQoL in CRC survivors in a systematic review [27]. Using the World Health Organization’s International Classification of Functioning, Disability and Health (WHO-ICF) as guiding framework, candidate predictors were mapped across relevant biopsychosocial domains of health and functioning and classified according to their strength of evidence [27]. The systematic review served as evidence base for selecting relevant candidate predictors to be used for the initial development of risk prediction models for HRQoL in CRC survivors. Models should preferably also be internally validated during the model development phase, which means testing the initial model for reproducibility [17, 25, 26]. Subsequently, during the second step of prediction research, the predictive performance of newly developed and internally validated models needs to be evaluated in populations other than the population used for model development (external validation) to assess the generalizability of prediction models [25, 26]. Finally, before implementation of prediction models in clinical practice, the presentation (e.g. as a risk score) and clinical impact of externally validated models should ideally be evaluated by testing whether their application in practice leads to improved patient outcomes, such as HRQoL [25, 26].

In the present study, as a first step towards use of risk prediction models for HRQoL in oncology practice, multivariable prediction models to estimate the 1-year risk of low HRQoL in multiple domains were developed and internally validated in a large prospective cohort of long-term CRC survivors. We primarily aimed to develop well-performing internally valid prognostic models for separate HRQoL domains, based on a comprehensive set of evidence-based a priori defined biopsychosocial predictors. A secondary goal was to build models that are easy for clinical practice, and can be used to prevent low future HRQoL in at-risk CRC survivors.

## Methods

### Study population

**Fig. 1:**
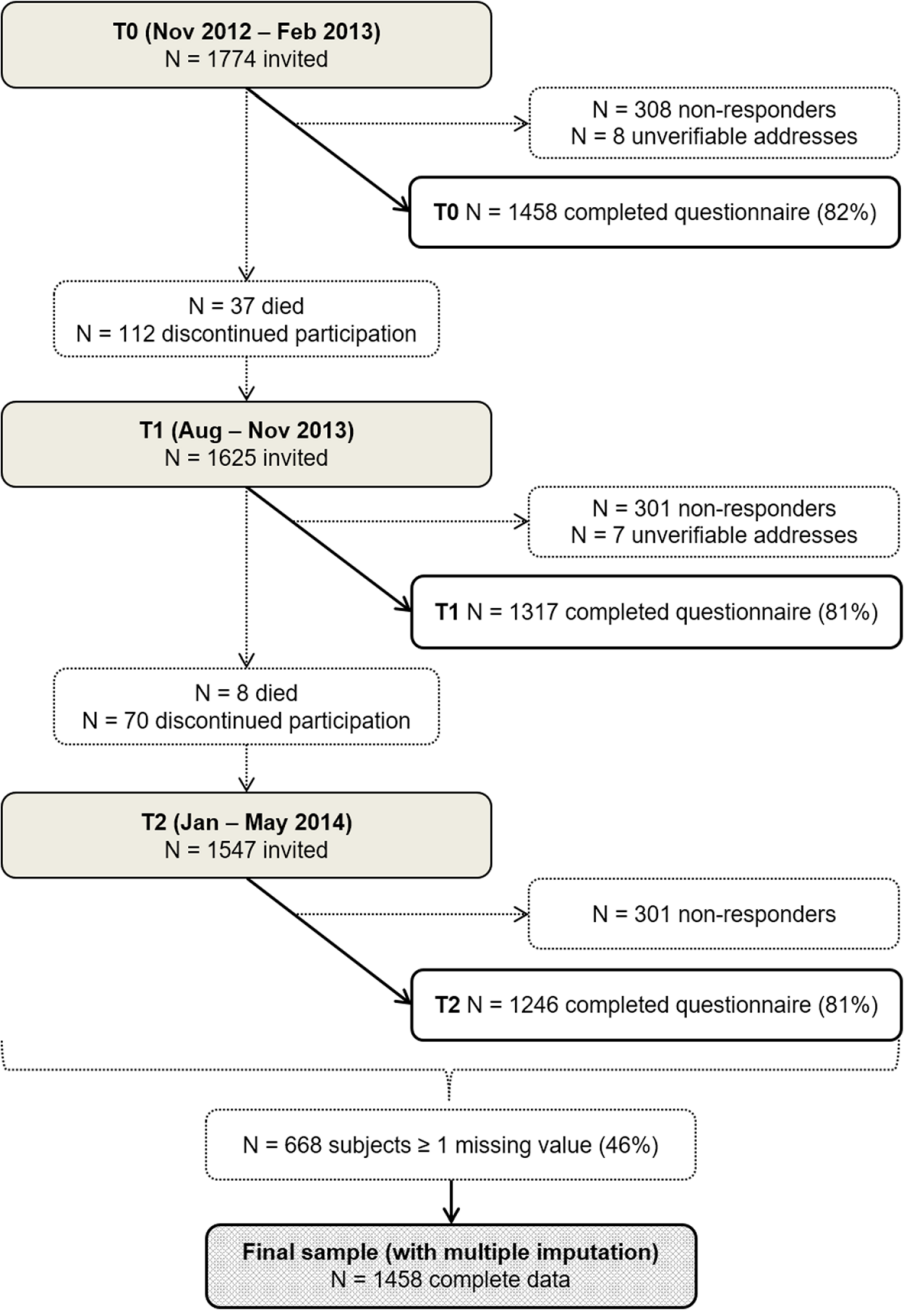
Flowchart of participant recruitment and inclusion at three consecutive waves (T0, T1 and T2) of the prospective cohort study in colorectal cancer survivors from the PROFILES registry

Data was used of stage I–IV CRC survivors participating in a prospective cohort study within the Patient Reported Outcomes Following Initial Treatment and Long-Term Evaluation of Survivorship (PROFILES) registry [28]. PROFILES is linked to the Netherlands Cancer Registry that routinely collects information from all newly diagnosed cancer patients in The Netherlands. The study was conducted according to the Declaration of Helsinki guidelines and approved by a certified local medical ethics committee, and written informed consent was obtained from all subjects before participation. Details of the data collection have previously been reported [28]. In short, CRC survivors participating in the prospective cohort study were asked to complete surveys with self-administered questionnaires, either online or on paper, in yearly waves from 2010 onwards. For the present analyses, we used data from three consecutive waves conducted between 2012 and 2014. Data from the first two waves (T0 and T1), which for individual participants was completed within a period of approximately 6 months, was considered as study baseline and used for assessment of candidate predictors. Data from the third wave (T2), which was completed for individual participants approximately 1 year after the first wave, was considered as follow-up for prediction of HRQoL. More details and timing of the three waves are shown in Fig. 1. All subjects who responded at the first wave (T0) were included in the present analyses (*N* = 1458).

### Data collection

#### Health-related quality of life

HRQoL was measured at T0 and T2 with the European Organization for Research and Treatment of Cancer Quality of life Questionnaire - Core 30 (EORTC QLQ-C30, Version 3.0) [29]. Seven subscales of this validated cancer-specific questionnaire were used for assessing the following HRQoL domains: global QoL; cognitive, emotional, physical, role, and social functioning; and fatigue. For every subscale a sum score was calculated ranging from 0 to 100 points, with higher scores on the global QoL and functioning scales representing better HRQoL and functioning, and higher scores on the fatigue scale representing worse fatigue [29]. Our goal was to develop prediction models for estimating the risk of having low HRQoL at follow-up (T2). Since interpretation of an individual’s continuous score on one or more of the HRQoL subscales of the EORTC QLQ-C30 is difficult in regard to risk prediction, the scores of the separate HRQoL subscales were dichotomized into low vs. normal/high scores for the purpose of developing the prediction models to estimate the risk of low HRQoL. Cut-offs to dichotomize the subscale scores of the separate HRQoL domains were determined based on previously published medium-to-large minimally important deteriorations (MID) in the EORTC QLQ-C30 subscales [30]. Accordingly, individuals were classified as having low HRQoL within each domain when having a subscale score at T2 ≥ 1 MID below the group average subscale score at T0; otherwise they were classified as having normal/high HRQoL. In this way, the low HRQoL group was comprised of individuals who either reported a constantly low HRQoL score at both T0 and T2, or who experienced a clinically relevant deterioration from a normal/high HRQoL score at T0 to a low HRQoL score at T2 (Table 1).

**Table 1:** Health-related quality of life (HRQoL) domains at baseline and follow-up of the entire study population in the non-imputed dataset (*N* = 1458)

	HRQoL at T0		HRQoL at T2		Dichotomized HRQoL groups			HRQoL changes from T0 to T2	
	N	Mean (SD)	N	Mean (SD)	MID ^b^	Cut-off ^c^	Low HRQoL at T2 N (%) ^c^	Consistently low HRQoL N (%) ^d^	Deteriorating HRQoL N (%) ^d^
Global quality of life ^a^	1429	78.1 (17.2)	1170	78.3 (17.2)	10	68.1	341 (23.4%)	205 (60.1%)	129 (37.8%)
Cognitive Functioning ^a^	1426	85.5 (19.4)	1167	86.6 (18.7)	7	78.5	229 (15.7%)	159 (69.4%)	66 (28.8%)
Emotional Functioning ^a^	1421	87.3 (18.5)	1164	88.2 (17.1)	12	75.3	270 (18.5%)	169 (62.6%)	93 (34.4%)
Physical Functioning ^a^	1431	81.7 (19.4)	1168	81.7 (19.1)	10	71.7	278 (19.1%)	197 (70.9%)	78 (28.1%)
Role Functioning ^a^	1423	81.9 (25.8)	1168	82.9 (25.1)	14	67.9	371 (25.4%)	238 (64.2%)	128 (34.5%)
Social Functioning ^a^	1419	88.2 (20.0)	1165	89.1 (20.1)	11	77.2	233 (16.0%)	133 (57.1%)	95 (40.8%)
Fatigue ^a^	1421	20.2 (22.2)	1157	20.5 (22.3)	10	30.2	349 (23.9%)	220 (63.0%)	118 (33.8%)

Footnotes:

^a^Higher scores on global QoL and functioning domains represent better HRQoL, whereas higher scores on fatigue represent worse fatigue complaints. All domains scores can range from 0 to 100 points

^b^Minimal important deterioration (MID) in EORTC QLQ-C30 domains published by Cocks et al. for each subscale [18]

^c^Persons were classified as having ‘low HRQoL’ when their T2 score differed by ≥1 MID from the T0 group mean (*below* mean for global quality of life and functioning domains; *above* mean for fatigue)

^d^Persons had consistently low HRQoL when both their T0 and T2 scores were ≥ 1 MID above/below the T0 group mean. Persons had deteriorating HRQoL when they decreased from normal/high HRQoL at T0 to a T2 score ≥ 1 MID below/above the T0 group mean (*below* mean for global quality of life and functioning domains; *above* mean for fatigue). The number of subjects with consistently low and deteriorating HRQoL do not add up to the total number in the low HRQoL group due to missings at T0

#### Candidate predictors

Using our previously published biopsychosocial WHO-ICF framework [27], a comprehensive set of sociodemographic, clinical, lifestyle, and psychological factors was selected as candidate predictors, including both non-modifiable and modifiable variables (see Supplementary Figure 1). The majority of candidate predictors was measured at the first wave (T0), except for certain lifestyle factors that were measured in a subsequent wave approximately 6 months later (T1).

##### Sociodemographic factors

Sociodemographic predictors included age, sex, current marital status (married or cohabiting, yes/no), and current work status (yes/no). Socio-economic status (SES) was categorized into low, medium or high, based on individual fiscal data from the year 2000 on the economic value of homes and household incomes, aggregated per postal code [31].

##### Clinical factors

Comorbidities were assessed with the adjusted Self-Administered Comorbidity Questionnaire (SCQ) [32], and categorized into 0, 1, or ≥ 2 comorbidities. Clinical data related to the patient’s history of CRC included the date of diagnosis, tumor site (colon or rectum), tumor stage (I-IV), and treatments received in addition to surgery (chemotherapy and/or radiotherapy). The presence of a stoma was assessed with the CRC-specific CR38 module of the EORTC QLQ [33].

Symptom scales and single items of the EORTC QLQ-C30 and CR38 were used to assess cancer-related symptoms, including fatigue, stoma-related complaints (for persons without a stoma, missing values were imputed with a ‘0’ for ‘no complaints’), pain, micturition, and chemotherapy-related side effects. Baseline fatigue scores were entered into all models as predictor based on strong evidence for its relevance as a HRQoL predictor [27, 34]. The separate subscale scores of nausea/vomiting, constipation, diarrhea, defecation problems, and gastrointestinal problems were summed into a total score for ‘gastrointestinal symptoms’.

##### Lifestyle factors

As measures of body fatness, body mass index (BMI, kg/m^2^) was calculated from self-reported height and weight at T0, and self-assessed waist circumference (cm) at T1. Current smoking status (y/n) was assessed by self-report at T0, whereas alcohol consumption, physical activity, and fruit, vegetable and total meat consumption were collected at T1 by validated questionnaires. Based on the 2007 World Cancer Research Fund/American Institute for Cancer Research (WCRF/AICR) lifestyle recommendations [35], participants were categorized into non-drinkers, mild-moderate drinkers (≤1 drinks/day for women and ≤ 2 drinks/day for men), or heavy drinkers (> 1 drink/day for women and > 2 drinks/day for men). Physical activity was assessed by the Short QUestionnaire to ASsess Health-enhancing physical activity (SQUASH) [36]. Total time spent in moderate-to-vigorous intensity physical activity (MVPA, min/day) was calculated [36, 37], on the basis of which adherence (y/n) to the Dutch physical activity standard was determined (i.e. MVPA ≥30 min/day on ≥5 days/week). Dietary intake was measured by an adapted version of the Dutch Healthy Diet–Food Frequency Questionnaire (DHD-FFQ) [38]. Adherence to the 2007 WCRF/AICR guidelines regarding fruit and vegetable intake and meat consumption [35] was defined as eating ≥5 portions of fruits and/or vegetables each day (y/n) and eating < 5 portions of meat per week (y/n).

##### Psychological factors

Separate scores for anxiety and depressive symptoms were calculated from the Hospital Anxiety and Depression Scale (HADS, range: 0–21 points), with higher scores indicating more symptoms [39]. Subscales of the Dutch 14-item Type D Personality Scale (DS-14) [40] were used to assess ‘Negative Affectivity’ (i.e. the tendency to experience negative emotions) and ‘Social Inhibition’ (i.e. the tendency to inhibit expression of emotions in social interaction) [41].

### Statistical analyses

Prior to analyses, incomplete data on candidate predictors and HRQoL outcomes was imputed with 50 multiple imputations using predictive mean matching in the *mice* package in R [42]. Multivariable logistic regression analyses were performed to develop separate prediction models for the seven HRQoL domains in the *rms* package in R [43]. Based on the previously developed WHO-ICF framework [27], 12 factors for which strong evidence regarding their potential importance as HRQoL predictors was available were entered into all models (shown in bold in Supplementary Figure 1): age, sex, socio-economic status, number of co-morbidities, time since diagnosis, stoma, BMI, physical activity, anxiety and depression scores, baseline fatigue and baseline HRQoL score of the specific domain. Additionally, in each of the 50 imputed datasets, other candidate predictors for which the evidence was considered weak-to-moderate or inconclusive [27] were tested for inclusion into the models by a backwards stepwise elimination procedure, using *P* < 0.1573 as cut-off for inclusion based on Akaike’s Information Criterion [44, 45]. Predictors were included in the final models when they were not eliminated from the models in ≥50% of the 50 imputed datasets [46]. Finally, regression coefficients from each imputed dataset were pooled using Rubin’s rules [47].

Measures of discrimination, calibration, overall performance, and classification were determined for each final model for the separate HRQoL domains. Discriminative ability describes how well a model can distinguish between individuals with low vs. normal/high HRQoL based on estimated risks, as quantified by the area under the Receiver Operator Characteristic curve (AUC, with AUC > 0.8 indicating good discrimination) [48]. Calibration is the agreement between predicted probabilities (risk) and observed relative frequencies (prevalence) of low HRQoL in the separate domains, as assessed by visual inspection of calibration plots showing agreement between predicted risk and observed prevalence of low HRQoL within deciles of predicted risk scores [49]. In addition, we used the Hosmer-Lemeshow goodness-of-fit test (H-L), with *P* > 0.05 indicating adequate calibration. To assess overall model performance, Nagelkerke’s R^2^ was determined as measure of predictive strength ranging between 0 and 1 with higher values indicating better performance, and Brier scores were determined as measures of model accuracy normally ranging between 0 and 0.25 with lower scores reflecting greater accuracy. Finally, for a range of predicted probabilities (10–80%), sensitivity and specificity of the models were determined as measures of classification, with sensitivity reflecting the probability that low HRQoL is correctly predicted in persons actually having low HRQoL (i.e. percentage of true-positive predictions given low HRQoL), and specificity reflecting the probability that normal/high HRQoL is correctly predicted in persons actually having normal/high HRQoL (i.e. percentage of true-negative predictions given no low HRQoL). We defined optimal threshold probabilities for the separate models based on high sensitivity (> 80%), as we considered false-negative predictions (i.e. misclassifying individuals with low HRQoL into the normal/high HRQoL group) more ‘harmful’ than false-positive predictions (i.e. misclassifying individuals with normal/high HRQoL into the low HRQoL group).

All final models were internally validated by bootstrapping using 1000 bootstrap samples to determine the degree of overfitting (i.e. models performing better in the development sample than in new samples consisting of other subjects) [44], yielding shrinkage factors for adjusting regression coefficients and adjusted model intercepts for incorporation into prediction formulas, and to assess optimism-corrected model performance measures [50, 51].

As sensitivity analyses, we reran the final models in the original non-imputed dataset to check if analyses yielded different conclusions after the multiple imputation as compared to complete-case analysis. Furthermore, we also performed backwards elimination procedures with less stringent *P*-values (*P* < 0.5) as cut-off for inclusion to assess whether relevant predictors were missed and affected model performance measures. In order to see the value of baseline HRQoL with regard to having low levels at the follow-up, we also ran models with only the respective baseline added, with the models excluding baseline, and compared the AUCs with the final models. All analyses were performed using R statistical software (R Foundation for Statistical Computing Platform 2016©, version 3.3.1). The Transparent Reporting of a multivariable prediction model for Individual Prognosis Or Diagnosis (TRIPOD) statement was used as guideline for analysis and reporting [25, 26].

## Results

### Population characteristics

Of the 1458 participants, 229 to 371 (16–25%) were categorized into the low HRQoL groups for the different domains, with the majority having consistently low HRQoL (57–71%, Table 1). Participants were on average 70 years of age and 5.1 years post-diagnosis, 43% was female, and 59 and 41% were diagnosed with colon or rectum cancer, respectively (Table 2). Complete data was available from 790 (54%) participants, whereas 668 participants (46%) had at least one missing value. Compared to participants with complete data, participants with incomplete data were more often female (48% vs. 39%), somewhat older (72 vs. 69 years), adhered less to physical activity guidelines (60% vs. 80%), and had somewhat lower HRQoL scores (3–11% more participants categorized into low HRQoL groups).

**Table 2:** Predictors measured at baseline and follow-up (T1) in the entire population in non-imputed dataset (*N* = 1458)

	N	N (%)
**Sociodemographic factors**		
Age (mean in years, SD)	1458	70.0 (9.3)
Sex (female)	1458	624 (42.8%)
Marital status (not married)	1439	322 (22.1%)
Work status (not working)	1415	1166 (80.0%)
Socio-economic status level	1419	
Low		263 (18.0%)
Medium		595 (40.8%)
High		561 (38.5%)
**Clinical Factors**		
Number of comorbidities	1381	
None		351 (24.1%)
1		429 (29.4%)
≥ 2		601 (41.2%)
Time since diagnosis (mean in years, SD)	1458	5.1 (0.1)
Tumor site (colon vs. rectum)	1458	861 (59.1%)
Tumor stage	1417	
I		446 (30.6%)
II		512 (35.1%)
III		418 (28.7%)
IV		41 (2.8%)
Stoma present (y/n)	1456	309 (21.2%)
Chemotherapy (y/n)	1458	431 (29.6%)
Radiotherapy (y/n)	1458	478 (32.8%)
Stoma-related complaints (mean, SD)	1448	3.9 (11.4)
Pain (mean, SD)	1426	15.9 (23.7)
Micturition problems (mean, SD)	1404	21.7 (17.6)
Chemotherapy-related side effects (mean, SD)	1407	10.0 (14.8)
Gastro-intestinal complaints (mean, SD)	1432	47.2 (51.8)
**Body composition and lifestyle factors**		
Body mass index (mean in kg/m^2^, SD)	1446	26.7 (4.1)
Waist circumference (mean in cm, SD) ^a^	1198	95.5 (14.8)
Smoking (y/n)	1420	141 (9.7%)
Alcohol consumption ^a^	1168	
Non-drinker		316 (21.7%)
Light-moderate drinker		560 (38.4%)
Heavy drinker		292 (20.0%)
Physical activity (adherence) ^a^	1242	903 (91.9%)
Fruit and vegetables (non-adherence) ^a^	1207	579 (39.7%)
Meat consumption (non-adherence) ^a^	1215	598 (41.0%)
**Psychological factors**		
Anxiety symptoms (mean, SD)	1412	4.3 (3.5)
Depressive symptoms (mean, SD)	1419	4.3 (3.5)
Negative affectivity (mean, SD)	1402	6.6 (5.9)
Social inhibition (mean, SD)	1409	7.6 (5.9)

^a^ Predictors measured at T1

### Prediction model development and internal validation

In the different prediction models for the seven separate HRQoL domains, 14 to 18 predictors were included in total, of which 12 predictors were entered into all models (or 11 for the model with fatigue as outcome) and 2 to 6 additional predictors were selected based on the backwards elimination procedure. Table 3 shows the intercepts and pooled regression coefficients of the predictors after correction for the shrinkage factors. Even though associations of individual predictors with the outcomes are not of primary importance when developing and evaluating performance of risk prediction models, optimism-corrected odds ratios are presented in Supplementary Table 1 to provide an indication of the magnitude and direction of the relations of each predictor with the separate HRQoL outcomes.

**Table 3:** Regression coefficients of the included predictors of the seven prediction models for health-related quality of life (HRQoL), after internal validation and shrinkage

	Global quality of life^c^	Cognitive Functioning^c^	Emotional Functioning^c^	Physical Functioning^c^	Role Functioning^c^	Social Functioning^c^	Fatigue^c^
**Intercept**	−0.33	2.37	−0.62	1.20	−1.50	−0.71	−3.96
**Included forced entry predictors**^**a**^							
Age (years)	0.02	0.00	0.02	0.05	0.03	0.02	0.01
Sex (ref = male)	0.27	− 0.16	0.11	0.42	0.15	−0.02	0.20
Socio-economic status (ref = high)							
Medium	0.22	0.13	0.14	−0.06	0.21	0.06	−0.11
Low	0.00	0.17	−0.03	−0.19	− 0.05	−0.20	− 0.42
Number of co-morbidities (ref = none)							
1	−0.14	0.17	0.06	0.18	0.06	0.22	0.12
≥ 2	0.10	−0.10	0.23	0.49	0.32	0.47	0.29
Time since diagnosis (years)	0.02	−0.04	0.03	−0.01	− 0.03	−0.01	0.00
Stoma presence (ref = no)	−0.18	0.00	0.11	−0.02	0.40	0.28	−0.21
Body mass index (kg/m^2^)	0.02	−0.01	−0.05	0.01	−0.01	0.00	−0.01
Physical activity (ref = non-adherence)	−0.36	0.02	−0.06	−0.64	− 0.44	−0.25	− 0.45
Anxiety symptom score	0.02	−0.01	0.14	0.03	0.05	−0.01	0.04
Depressive symptom score	0.08	0.07	0.03	0.03	0.04	0.08	0.05
Baseline fatigue	0.01	0.01	0.01	0.01	0.01	0.01	0.05
Baseline HRQoL (specific per domain)	−0.05	−0.06	−0.04	−0.10	− 0.03	−0.04	–
**Included predictors based on backwards selection**^**b**^							
Chemotherapy (ref = no)	0.25					−0.35	
Radiotherapy (ref = no)	0.27						
Tumour stage (ref = stage I)							
Stage II						−0.11	
Stage III						0.52	
Stage IV						0.50	
Working status (ref = no)			0.48				
Smoking (ref = no)	0.36		0.41	0.66		0.56	0.81
Social inhibition score			−0.03		0.02		
Negative affectivity score		0.03	0.06			0.04	
Micturition score		0.01					
Chemotherapy side effects score	0.01		0.01			0.01	0.01
Stoma complaints score	0.01			0.02			0.02
Gastrointestinal complaints sum score						0.01	0.00
Pain score	0.01				0.01		0.01
Meat consumption adherence (ref = yes)			0.22				

Footnotes:

^a^ Twelve candidate predictors were forced into each model, as there was strong evidence for their association with HRQoL in a systematic review [19]

^b^ Candidate predictors for which moderate or weak evidence was found, were selected with backwards selection procedures using Akaike’s Information Criterion (*p* < 0.1573). The following candidate predictors were not included in any of the models: tumor localization, marital status, fruit and vegetable consumption, alcohol consumption and waist circumference

^c^ Regression coefficients display the ln (odds) change in outcome, but no standard errors could be calculated after shrinkage; Formula for the probability of having low HRQoL = 1 / (1 + exp.[− Linear predictor]);

Linear predictor = intercept + sum of (predictor * regression coefficient)

**Fig. 2:**
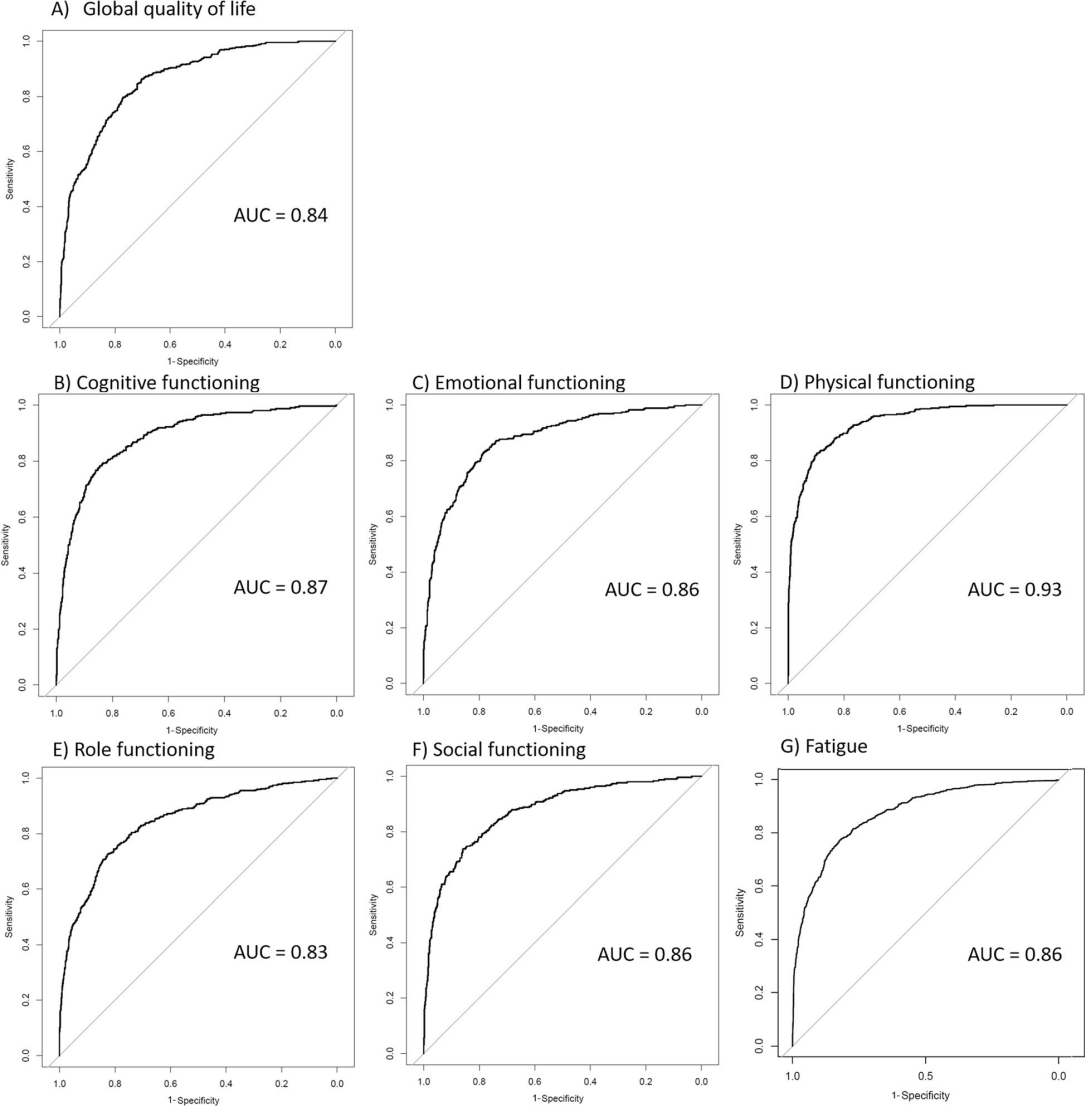
For each health-related quality of life domain we show the area under the Receiver Operator Characteristic curve (AUC, with AUC > 0.8 indicating good discrimination)

All model performance measures are shown in detail in Supplementary Table 2. Internal validation yielded shrinkage factors ranging between 0.89 and 0.91 for the separate models. The optimism-corrected AUC values ranged between 0.83 and 0.93, which are also shown together with the ROC curves in Fig. 2. Nagelkerke’s R^2^ values ranged between 0.40 and 0.63, and Brier scores between 0.09 and 0.15. Calibration of the models was good, as indicated by calibration plots showing good agreement between actual and predicted probabilities for all models (Supplementary Figure 2). Additionally, all Hosmer-Lemeshow goodness-of-fit tests were non-significant for all HRQoL domains (*P*-values ranging between 0.32 and 0.95). Graphs with sensitivity and specificity plotted for the separate models across a range 10 to 80% predicted risk of low HRQoL showed that a sensitivity of 80% or higher was reached when predicted risks between 10 and 30% were used a cut-off for a positive prediction, i.e. classification of an individual into the low HRQoL group based on the predicted risk score (Supplementary Figure 3). Overall, the prediction model with physical functioning as outcome was the model that showed the best performance.

### Sensitivity analyses

Sensitivity analyses demonstrated that the final models were robust, as they performed similarly in the imputed and the original non-imputed datasets, yielding comparable AUC values (AUC range: 0.85–0.94, data not shown). In addition, AUC values also did not change when less stringent backward selection criteria (*P* < 0.5) were used for model development (AUC range: 0.85–0.94, data not shown). The AUCs of models were slightly smaller when they contained only baseline HRQoL (AUC range: 0.80–0.92), or without any baseline HRQoL (AUC range: 0.78–0.88, as shown in Supplementary Table 3).

## Discussion

Risk prediction models for seven HRQoL domains in long-term CRC survivors were developed and internally validated, containing a comprehensive set of evidence-based biopsychosocial predictors and showing good to excellent model performance. These models are ready for external validation in other cohorts of CRC survivors, who are for instance situated closer to diagnosis and treatment. This would be to evaluate whether they are generalizable and could be useful tools in oncology practice for identifying individual CRC survivors at risk of experiencing low HRQoL approximately one year after the moment of prediction. Thus, use of the prediction models can enable selection of high-risk individuals who might benefit from interventions aimed at improving or safeguarding their future HRQoL.

As the first important step in prediction research, this large-scale study has provided internally valid prediction models for estimating the 1-year risk of having low HRQoL. Firstly, these models have lifestyle and psychosocial predictors included that are selected based on evidence from previous association studies summarized in a systematic review [27]. When we ran the models with only the respective baseline HRQoL values, their AUCs were relatively high, confirming the prior expectation that baseline HRQoL alone is an important predictor that should be included in the models. Nevertheless, the AUCs increase when the other predictors are included, indicating improved predictions. Moreover, the table shows that the other predictors do have adequate predictive power, as shown by the AUC results of the models without baseline HRQoL. The associations between HRQoL and the specific predictors were not part of our scope, and therefore, we should be cautious with a causal interpretation of relations between predictors and outcomes based on prediction models. Even though the developed risk prediction models included partially overlapping predictors, the models for each of the seven HRQoL domains had their own unique features and contributions to the risk estimation. Moreover, low to moderate correlations observed among the HRQoL domains (Spearman’s rho: 0.3–0.5; data not shown) indicated that the low HRQoL groups for the separate domains were not comprised of the same CRC survivors, reflecting that HRQoL is a multi-dimensional construct consisting of different aspects that are covered by the separate domains.

The predictive power of all 7 models was good to excellent. The models were found to generate accurate risk predictions that enabled good discrimination between individual CRC survivors who did or who did not experience low HRQoL scores in the future. Further, it was found that optimal probability thresholds for good classification of low vs. normal/high HRQoL based on predicted risks mostly ranged between 10 and 30%. If predicted risks within this range were used as cut-off for positive predictions (i.e. classification of an individual survivor as being at risk of low HRQoL), the sensitivities of the models were > 80% which is considered high. We preferred a high sensitivity of the models over a high specificity, because we did not want to misclassify many survivors with low HRQoL (false-negatives) who could benefit from interventions targeted at improving their future HRQoL. We accepted lower specificity of the models (i.e. increased chance of false-positive predictions) since we deemed providing unnecessary HRQoL interventions, which are not invasive or hazardous, less problematic than not providing necessary HRQoL interventions.

For the current study, long-term CRC survivors participating in an ongoing prospective cohort study were selected. Two third of the survivors classified into the low HRQoL group at study follow-up also had low HRQoL scores at baseline, indicative of a consistently low level of HRQoL. Nevertheless, a substantial percentage of the CRC survivors showed a clinically relevant deterioration of HRQoL scores over the approximately 1-year study period, which is rather striking when considering that the CRC survivors were on average five years after diagnosis. Larger changes in HRQoL are expected closer to diagnosis and treatment [52], which may be a more relevant time frame for prediction and taking preventive action. Therefore, the next step should be to externally validate the developed models in other CRC survivor populations to determine whether their predictive abilities are transferable to a more immediate post-treatment time frame. Subsequently, the benefit of these models should also be evaluated in so-called clinical impact studies to assess whether risk prediction is of added value and can contribute to improving HRQoL outcomes in oncology practice. This final and important step of prediction research is often overlooked. For instance, several prediction models have been developed, and to a lesser extent externally validated, for estimating probabilities of survival in CRC patients to be used when considering different treatment options [18–20]. One recently published prediction model for survival has even presented an online tool for use in clinical practice during the treatment phase [20]. However, none of these previously developed models for survival have been evaluated in clinical impact studies to assess whether their application actually can improve survival through improved tailoring of treatments.

The present study has several strengths, including its large sample size, high response rate, and longitudinal design. In addition, sophisticated statistical methods were used that are currently recommended in the field of prediction modelling, such as multiple imputation and bootstrapping [26]. Furthermore, all predictors were selected from the literature based on previous evidence [27], thereby emphasizing theory-driven instead of data-driven predictor selection. Moreover, our study is novel as, to the best of our knowledge, no prediction models for estimating future HRQoL in CRC survivors after treatment are currently available. Both clinicians and CRC survivors could benefit from future implementation of such models in the form of, for example, online calculators or as add-ons to existing lifestyle and clinical guidelines (e.g. from WCRF/AICR [35, 53] and American Cancer Society [1]) that focus mostly on cancer prevention and survival but less on HRQoL.

Next to its strengths, the study also has some limitations. First, as already pointed out, the study participants were long-term CRC survivors on average five years after diagnosis, therefore representing a population that probably had relatively stabilized HRQoL. As mentioned, future external validation of these models is warranted in cohorts closer to diagnosis and treatment, when larger changes are expected in HRQoL. Second, we dichotomized the continuous HRQoL outcomes for ease of interpretation and risk estimation, which may have led to loss of information. The classification of survivors in the low and normal/high HRQoL groups at study follow-up was determined based on the mean HRQoL scores at the study baseline, and therefore population-dependent. However, we also incorporated previously reported cut-offs for HRQoL deteriorations to make the classifications more clinically relevant and generalizable [30]. Third, we defined the low HRQoL group as having low HRQoL at study follow-up, thereby not distinguishing between individuals with consistently low HRQoL or with deteriorated HRQoL over time. Though both groups of individuals would be eligible for interventions aimed at safeguarding their HRQoL, future studies could elaborate on the longitudinal course of HRQoL and on possible different characteristics of individuals at risk of having constant low levels of HRQoL or deteriorating levels of HRQoL. Fourth, regardless of the large sample size, models had a range of 8 to 13 events per predictor, and some models had less than the recommended ≥10 events per predictor for model development and less than 250 events for the internal validation [26], which might have impacted the stability of the performance measures. In a recently published tool to assess risk of bias in prediction model studies, Moons et al. state that development studies should have more than 20 events per predictor, and more than 100 events for the validations [54]. Moreover, we did not apply any interaction terms at the development of these models for terms like age or BMI. Most of the included participants were older (mean age = 70.0 years; SD = 9.3), as most CRC survivors are in the practice too. Also, BMI may be interesting to look at when we distinguish between underweight and overweight, but also there was not that much difference (mean BMI = 26.7; SD = 4.1). We are aware of the multiple techniques available for prediction modelling in addition to regression analyses (e.g., machine learning techniques such as random forests, neural networks), but we now used regression modelling, as this is how the majority of the models is developed. Moreover, advanced techniques are not always superior [55], and it remains transparent, reproducible and understandable for clinicians and researchers. Lastly, imputation of missing values might have introduced bias if the missings were not random. Although this assumption is untestable, multiple imputation was used as the currently recommended strategy for imputing missing data with the least risk of bias [26, 54].

## Conclusion

To our knowledge, this is the first study that developed and internally validated prediction models for HRQoL in CRC survivors, focusing on estimating the 1-year risk of low HRQoL in multiple domains (global QoL; cognitive, emotional, physical, role, and social functioning; and fatigue). The models showed good to excellent predictive performance for identifying CRC survivors who are at increased risk of experiencing low HRQoL in the future and who are eligible for preventive interventions. The included set of biopsychosocial predictors, of which several are modifiable, have been significantly associated with HRQoL in CRC survivors in the literature. In the future, external validation and a clinical impact evaluation are needed before these models should be used for decision making. As there is often a lack of time during oncological consultations to discuss HRQoL problems, prediction models can enhance efficient communication with patients and shared decision-making. The developed models are important as a first step towards future implementation of risk prediction tools in oncology practice specifically aimed at the HRQoL of the growing population of CRC survivors.

## Supplementary information

**Additional file 1: Supplemental Figure S1.** Predictors mapped across domains of the World Health Organization’s International Classification of Functioning, Disability and Health (WHO-ICF) framework, and selected for the prediction models based on previous evidence: 12 fixed predictors entered into all models (in bold with arrows) and 18 candidate predictors selected for backwards elimination. Some candidate predictors were measured at T1 instead of T0; this is indicated between brackets.

**Additional file 2: Supplemental Figure S2.** Calibration plots for seven health-related quality of life (HRQoL) domains.

**Additional file 3: Supplemental Figure S3.** Classification measures of the models for the seven health-related quality of life (HRQoL) domains with various threshold probabilities (10–80%): sensitivity (probability of true-positive prediction given low HRQoL; black line) and specificity (probability of true-negative prediction given no low HRQoL; dotted line). Grey boxes highlight threshold probabilities that correspond to sensitivity > 80%.

**Additional file 4: Supplemental Table S1.** Odds ratios of included predictors of the seven prediction models for health-related quality of life (HRQoL) after internal validation.

**Additional file 5: Supplemental Table S2.** Model performance measures of the seven prediction models for health-related quality of life. Performance measures of the original models and the models after internal validation are presented.

**Additional file 6: Supplemental Table S3.** Sensitivity analyses of the seven prediction models for health-related quality of life (HRQoL), with only respective baseline HRQoL values, without baseline HRQoL and with the complete models.

## Data Availability

The datasets used and/or analysed during the current study are available from the corresponding author on reasonable request.

## Abbreviations

AUC: Area under the Receiver Operator Characteristic curve
BMI: Body mass index
CRC: Colorectal cancer
DHD-FFQ: Dutch Healthy Diet–Food Frequency Questionnaire
DS-14: Dutch 14-item Type D Personality Scale
EORTC QLQ-C30: European Organization for Research and Treatment of Cancer Quality of life Questionnaire - Core 30
HADS: Hospital Anxiety and Depression Scale
H-L: Hosmer-Lemeshow goodness-of-fit test
HRQoL: Health-related quality of life
MID: Minimally important deteriorations
MVPA: Moderate-to-vigorous intensity physical activity
PROFILES: Patient Reported Outcomes Following Initial Treatment and Long-Term Evaluation of Survivorship
QoL: Quality of life
SES: Socio-economic status
SQUASH: Short QUestionnaire to ASsess Health-enhancing physical activity
TRIPOD: Transparent Reporting of a multivariable prediction model for Individual Prognosis Or Diagnosis
WCRF/AICR: World Cancer Research Fund/American Institute for Cancer Research
WHO-ICF: World Health Organization’s International Classification of Functioning, Disability and Health
