# Supplementary material for: Development and internal validation of prediction models for colorectal cancer survivors to estimate the 1-year risk of low health-related quality of life in multiple domains
Source: BMC Med Inform Decis Mak. 2020 Mar 12;20:54. doi: 10.1186/s12911-020-1064-9 (PMC7068880; doi:10.1186/s12911-020-1064-9)
Supplement: Supplementary file 2 — Additional file 2: Supplemental Figure S2. Calibration plots for seven health-related quality of life (HRQoL) domains. [file 12911_2020_1064_MOESM2_ESM.docx]

**BMC Medical Informatics and Decision Making - Supplementary Figures**

**Development and internal validation of prediction models for colorectal cancer survivors to estimate the 1-year risk of low health-related quality of life in multiple domains**

**Authors:**

Dóra Révész^1,2^, Sander M.J. van Kuijk^3^, Floortje Mols^2,4^, Fränzel J.B. van Duijnhoven^5^, Renate M. Winkels^6^, Huub Hoofs^7^, IJmert Kant^7^, Luc J. Smits^7^, Stéphanie O. Breukink^8^, Lonneke V. van de Poll-Franse^3,4,9^, Ellen Kampman^5^, Sandra Beijer^4^, Matty P. Weijenberg^1^, Martijn J.L. Bours^1^

**Author affiliations**

^1^ Department of Epidemiology, GROW – School for Oncology and Developmental Biology, Maastricht University, P. Debyeplein 1, 6200 MD Maastricht, the Netherlands

^2^ CoRPS – Center of Research on Psychology in Somatic diseases, Department of Medical and Clinical Psychology, Tilburg University, Warandelaan 2, 5037 AB Tilburg, the Netherlands

^3^ Clinical Epidemiology and Medical Technology Assessment, Maastricht University Medical Centre+, P. Debyelaan 25, PO Box 5800, Maastricht 6202 AZ, the Netherlands

^4^ Netherlands Comprehensive Cancer Organisation (IKNL), Godebaldkwartier 419, 3511 DT Utrecht, the Netherlands

^5^ Division of Human Nutrition, Wageningen University & Research, Stippeneng 4, 6708 WE Wageningen, the Netherlands

^6^ Department of Public Health Sciences, Penn State Cancer Institute, 500 University Drive Hershey, PA 17033, USA

^7^ Department of Epidemiology, CAPHRI School for Public Health and Primary Care, Faculty of Health, Medicine and Life Sciences, Maastricht University, P. Debyeplein 1, 6200 MD Maastricht, the Netherlands

^8^ Department of Surgery, Maastricht University Medical Centre, P. Debyelaan 25, 6229 HX Maastricht, the Netherlands

^9^ Department of Psychosocial Oncology and Epidemiology, Netherlands Cancer Institute, Plesmanlaan 121, 1066 CX Amsterdam, the Netherlands

**Corresponding author:**

Dóra Révész, PhD

Department of Epidemiology, GROW – School for Oncology and Developmental Biology, Maastricht University, P. Debyeplein 1, 6200 MD Maastricht, the Netherlands

[Dora.Revesz@maastrichtuniversity.nl](mailto:Dora.Revesz@maastrichtuniversity.nl) / [D.Revesz@uvt.nl](mailto:D.Revesz@uvt.nl)

T: +31 043 388 2903

F: +31 043 388 4128**Supplementary Figure 2.** Calibration plots for seven health-related quality of life (HRQoL) domains.

**
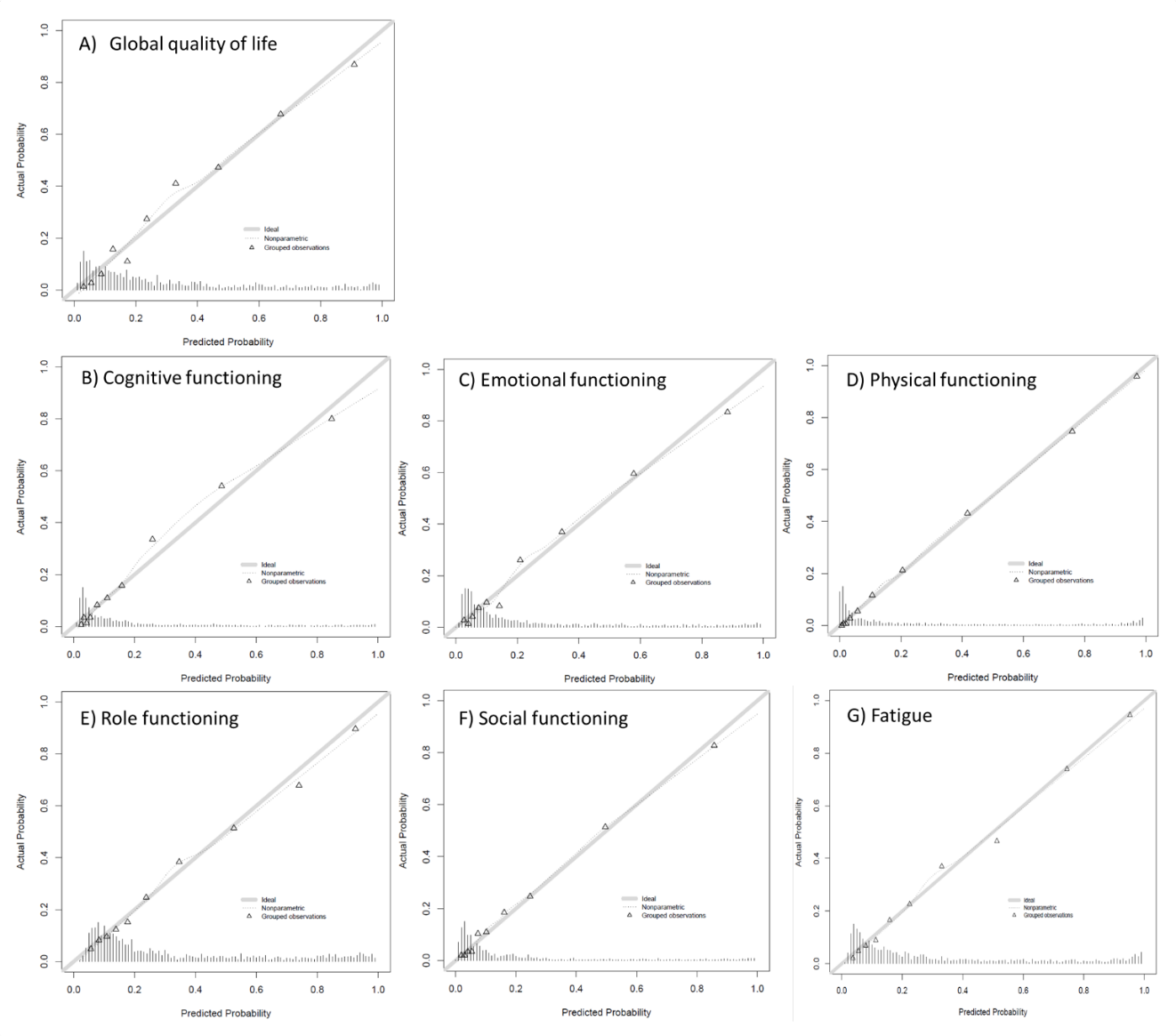
**
