# Supplementary material for: Development and internal validation of prediction models for colorectal cancer survivors to estimate the 1-year risk of low health-related quality of life in multiple domains
Source: BMC Med Inform Decis Mak. 2020 Mar 12;20:54. doi: 10.1186/s12911-020-1064-9 (PMC7068880; doi:10.1186/s12911-020-1064-9)
Supplement: Supplementary file 4 — Additional file 4: Supplemental Table S1. Odds ratios of included predictors of the seven prediction models for health-related quality of life (HRQoL) after internal validation. [file 12911_2020_1064_MOESM4_ESM.docx]

**BMC Medical Informatics and Decision Making - Supplementary Figures**

**Development and internal validation of prediction models for colorectal cancer survivors to estimate the 1-year risk of low health-related quality of life in multiple domains**

**Authors:**

Dóra Révész^1,2^, Sander M.J. van Kuijk^3^, Floortje Mols^2,4^, Fränzel J.B. van Duijnhoven^5^, Renate M. Winkels^6^, Huub Hoofs^7^, IJmert Kant^7^, Luc J. Smits^7^, Stéphanie O. Breukink^8^, Lonneke V. van de Poll-Franse^3,4,9^, Ellen Kampman^5^, Sandra Beijer^4^, Matty P. Weijenberg^1^, Martijn J.L. Bours^1^

**Author affiliations**

^1^ Department of Epidemiology, GROW – School for Oncology and Developmental Biology, Maastricht University, P. Debyeplein 1, 6200 MD Maastricht, the Netherlands

^2^ CoRPS – Center of Research on Psychology in Somatic diseases, Department of Medical and Clinical Psychology, Tilburg University, Warandelaan 2, 5037 AB Tilburg, the Netherlands

^3^ Clinical Epidemiology and Medical Technology Assessment, Maastricht University Medical Centre+, P. Debyelaan 25, PO Box 5800, Maastricht 6202 AZ, the Netherlands

^4^ Netherlands Comprehensive Cancer Organisation (IKNL), Godebaldkwartier 419, 3511 DT Utrecht, the Netherlands

^5^ Division of Human Nutrition, Wageningen University & Research, Stippeneng 4, 6708 WE Wageningen, the Netherlands

^6^ Department of Public Health Sciences, Penn State Cancer Institute, 500 University Drive Hershey, PA 17033, USA

^7^ Department of Epidemiology, CAPHRI School for Public Health and Primary Care, Faculty of Health, Medicine and Life Sciences, Maastricht University, P. Debyeplein 1, 6200 MD Maastricht, the Netherlands

^8^ Department of Surgery, Maastricht University Medical Centre, P. Debyelaan 25, 6229 HX Maastricht, the Netherlands

^9^ Department of Psychosocial Oncology and Epidemiology, Netherlands Cancer Institute, Plesmanlaan 121, 1066 CX Amsterdam, the Netherlands

**Corresponding author:**

Dóra Révész, PhD

Department of Epidemiology, GROW – School for Oncology and Developmental Biology, Maastricht University, P. Debyeplein 1, 6200 MD Maastricht, the Netherlands

[Dora.Revesz@maastrichtuniversity.nl](mailto:Dora.Revesz@maastrichtuniversity.nl) / [D.Revesz@uvt.nl](mailto:D.Revesz@uvt.nl)

T: +31 043 388 2903

F: +31 043 388 4128

| **Supplementary Table 1**: Odds ratios of included predictors of the seven prediction models for health-related quality of life (HRQoL) after internal validation. | | | | | | | | | |
| --- | --- | --- | --- | --- | --- | --- | --- | --- | --- |
|  | | | **Global quality**  **of life ^c^** | **Cognitive Functioning ^c^** | **Emotional Functioning ^c^** | **Physical Functioning ^c^** | **Role**  **Functioning ^c^** | **Social Functioning ^c^** | **Fatigue ^c^** |
| **Included forced entry predictors ^a^** | | | | | | | | | |
| Age (years) | | | 1.02 | 1.00 | 1.02 | 1.05 | 1.03 | 1.02 | 1.01 |
| Sex (ref=male) | | | 1.31 | 0.85 | 1.11 | 1.52 | 1.17 | 0.98 | 1.22 |
| Socio-economic status (ref=high) | | Medium | 1.24 | 1.14 | 1.14 | 0.94 | 1.23 | 1.06 | 0.90 |
|  |  | Low | 1.00 | 1.19 | 0.97 | 0.83 | 0.96 | 0.82 | 0.65 |
| Number of co-morbidities (ref=none) | | 1 | 0.87 | 1.18 | 1.07 | 1.20 | 1.07 | 1.25 | 1.12 |
|  |  | ≥2 | 1.11 | 0.90 | 1.25 | 1.63 | 1.38 | 1.60 | 1.34 |
| Time since diagnosis (years) | | | 1.02 | 0.96 | 1.03 | 0.99 | 0.97 | 0.99 | 1.00 |
| Stoma presence (ref=no) | | | 0.84 | 1.00 | 1.11 | 0.98 | 1.49 | 1.33 | 0.81 |
| Body mass index (kg/m^2^) | | | 1.02 | 0.99 | 0.96 | 1.01 | 0.99 | 1.00 | 0.99 |
| Physical activity  (ref=non-adherence) | | | 0.70 | 1.02 | 0.94 | 0.53 | 0.65 | 0.78 | 0.64 |
| Anxiety symptom score | | | 1.02 | 0.99 | 1.15 | 1.03 | 1.06 | 0.99 | 1.04 |
| Depressive symptom score | | | 1.09 | 1.07 | 1.03 | 1.03 | 1.04 | 1.08 | 1.06 |
| Baseline fatigue score | | | 1.01 | 1.01 | 1.01 | 1.01 | 1.01 | 1.01 | 1.05 |
| Baseline HRQoL score | | | 0.95 | 0.94 | 0.96 | 0.90 | 0.97 | 0.96 | - |
| **Included predictors based on backwards selection ^b^** | | | | | | | | | |
| Chemotherapy (ref=no) | | | 1.28 |  |  |  |  | 0.71 |  |
| Radiotherapy (ref=no) | | | 1.31 |  |  |  |  |  |  |
| Tumor stage (ref=stage I) | Stage II | |  |  |  |  |  | 0.90 |  |
|  | Stage III | |  |  |  |  |  | 1.68 |  |
|  | Stage IV | |  |  |  |  |  | 1.65 |  |
| Working status (ref=no) | | |  |  | 1.61 |  |  |  |  |
| Smoking (ref=no) | | | 1.43 |  | 1.50 | 1.94 |  | 1.75 | 2.25 |
| Social inhibition score | | |  |  | 0.97 |  | 1.02 |  |  |
| Negative affectivity score | | |  | 1.04 | 1.07 |  |  | 1.04 |  |
| Micturition score | | |  | 1.01 |  |  |  |  |  |
| Chemotherapy side effects score | | | 1.01 |  | 1.01 |  |  | 1.01 | 1.01 |
| Stoma complaints score | | | 1.01 |  |  | 1.02 |  |  | 1.02 |
| Gastrointestinal complaints score | | |  |  |  |  |  | 1.01 | 1.00 |
| Pain score | | | 1.01 |  |  |  | 1.01 |  | 1.01 |
| Meat consumption (ref=adherence) | | |  |  | 1.24 |  |  |  |  |
| **Footnotes**:  ^a^ Twelve predictors were forced into each model, as there was strong evidence for their association with HRQoL [26].  ^b^ Candidate predictors for which moderate or weak evidence was found, were selected with backwards selection procedures using Akaike’s Information Criterion (P<0.1573).  ^c^ Odds ratios are provided to give an estimation of the strength of each predictor, but no confidence intervals could be calculated after the shrinkage of regression coefficients. | | | | | | | | | |
