# Supplementary material for: Development and internal validation of prediction models for colorectal cancer survivors to estimate the 1-year risk of low health-related quality of life in multiple domains
Source: BMC Med Inform Decis Mak. 2020 Mar 12;20:54. doi: 10.1186/s12911-020-1064-9 (PMC7068880; doi:10.1186/s12911-020-1064-9)
Supplement: Supplementary file 5 — Additional file 5: Supplemental Table S2. Model performance measures of the seven prediction models for health-related quality of life. Performance measures of the original models and the models after internal validation are presented. [file 12911_2020_1064_MOESM5_ESM.docx]

**BMC Medical Informatics and Decision Making - Supplementary Figures**

**Development and internal validation of prediction models for colorectal cancer survivors to estimate the 1-year risk of low health-related quality of life in multiple domains**

**Authors:**

Dóra Révész^1,2^, Sander M.J. van Kuijk^3^, Floortje Mols^2,4^, Fränzel J.B. van Duijnhoven^5^, Renate M. Winkels^6^, Huub Hoofs^7^, IJmert Kant^7^, Luc J. Smits^7^, Stéphanie O. Breukink^8^, Lonneke V. van de Poll-Franse^3,4,9^, Ellen Kampman^5^, Sandra Beijer^4^, Matty P. Weijenberg^1^, Martijn J.L. Bours^1^

**Author affiliations**

^1^ Department of Epidemiology, GROW – School for Oncology and Developmental Biology, Maastricht University, P. Debyeplein 1, 6200 MD Maastricht, the Netherlands

^2^ CoRPS – Center of Research on Psychology in Somatic diseases, Department of Medical and Clinical Psychology, Tilburg University, Warandelaan 2, 5037 AB Tilburg, the Netherlands

^3^ Clinical Epidemiology and Medical Technology Assessment, Maastricht University Medical Centre+, P. Debyelaan 25, PO Box 5800, Maastricht 6202 AZ, the Netherlands

^4^ Netherlands Comprehensive Cancer Organisation (IKNL), Godebaldkwartier 419, 3511 DT Utrecht, the Netherlands

^5^ Division of Human Nutrition, Wageningen University & Research, Stippeneng 4, 6708 WE Wageningen, the Netherlands

^6^ Department of Public Health Sciences, Penn State Cancer Institute, 500 University Drive Hershey, PA 17033, USA

^7^ Department of Epidemiology, CAPHRI School for Public Health and Primary Care, Faculty of Health, Medicine and Life Sciences, Maastricht University, P. Debyeplein 1, 6200 MD Maastricht, the Netherlands

^8^ Department of Surgery, Maastricht University Medical Centre, P. Debyelaan 25, 6229 HX Maastricht, the Netherlands

^9^ Department of Psychosocial Oncology and Epidemiology, Netherlands Cancer Institute, Plesmanlaan 121, 1066 CX Amsterdam, the Netherlands

**Corresponding author:**

Dóra Révész, PhD

Department of Epidemiology, GROW – School for Oncology and Developmental Biology, Maastricht University, P. Debyeplein 1, 6200 MD Maastricht, the Netherlands

[Dora.Revesz@maastrichtuniversity.nl](mailto:Dora.Revesz@maastrichtuniversity.nl) / [D.Revesz@uvt.nl](mailto:D.Revesz@uvt.nl)

T: +31 043 388 2903

F: +31 043 388 4128

| **Supplementary Table 2**: Model performance measures of the seven prediction models for health-related quality of life. Performance measures of the original models and the models after internal validation are presented. | | | | | | | |
| --- | --- | --- | --- | --- | --- | --- | --- |
|  | **Global quality of life** | **Cognitive Functioning** | **Emotional Functioning** | **Physical Functioning** | **Role Functioning** | **Social Functioning** | **Fatigue** |
| **Original models** | | | | | | | |
| AUC  [95% CI] ^a^ | 0.86  [0.84 – 0.88] | 0.88  [0.86 – 0.91] | 0.88  [0.86 – 0.90] | 0.94  [0.92 – 0.95] | 0.85  [0.83 – 0.87] | 0.88  [0.86 – 0.90] | 0.88  [0.86 – 0.89] |
| Nagelkerke’s R^2^ ^b^ | 0.45 | 0.47 | 0.48 | 0.66 | 0.45 | 0.48 | 0.51 |
| Brier score ^c^ | 0.14 | 0.10 | 0.11 | 0.08 | 0.14 | 0.10 | 0.13 |
| H-L test p-value ^d^ | 0.32 | 0.42 | 0.66 | 0.93 | 0.87 | 0.95 | 0.82 |
| **Internally validated models** | | | | | | | |
| Shrinkage factor ^e^ | 0.90 | 0.89 | 0.90 | 0.91 | 0.91 | 0.89 | 0.91 |
| AUC ^a^ | 0.84 | 0.87 | 0.86 | 0.93 | 0.83 | 0.86 | 0.86 |
| Nagelkerke’s R^2 b^ | 0.41 | 0.43 | 0.43 | 0.63 | 0.42 | 0.44 | 0.47 |
| Brier score ^c^ | 0.14 | 0.11 | 0.12 | 0.09 | 0.15 | 0.11 | 0.13 |
| **Footnotes**:  ^a^ AUC = Area under the Receiver Operator Characteristic curve; 95% confidence intervals were calculated for original models only; AUC ≥ 0.80 good discrimination).  ^b^ Nagelkerke’s R^2^ is a measure of explained variance, ranging from 0 to 1 (higher is better).  ^c^ Brier score is a measure of model accuracy, ranging from 0 (perfect) to 0.25 (worthless accuracy).  ^d^ H-L = Hosmer-Lemeshow goodness-of-fit test is an indicator of calibration (agreement between observed and predicted values) and is calculated for original models only; P>0.05 represents well-calibrated model (i.e. non-significant disagreement between observed and predicted values).  ^e^ Shrinkage factors are calculated during the bootstrapping procedure to correct for overfitting. | | | | | | | |
