# Supplementary material for: Development and internal validation of prediction models for colorectal cancer survivors to estimate the 1-year risk of low health-related quality of life in multiple domains
Source: BMC Med Inform Decis Mak. 2020 Mar 12;20:54. doi: 10.1186/s12911-020-1064-9 (PMC7068880; doi:10.1186/s12911-020-1064-9)
Supplement: Supplementary file 6 — Additional file 6: Supplemental Table S3. Sensitivity analyses of the seven prediction models for health-related quality of life (HRQoL), with only respective baseline HRQoL values, without baseline HRQoL and with the complete models. [file 12911_2020_1064_MOESM6_ESM.docx]

**BMC Medical Informatics and Decision Making - Supplementary Figures**

**Development and internal validation of prediction models for colorectal cancer survivors to estimate the 1-year risk of low health-related quality of life in multiple domains**

**Authors:**

Dóra Révész^1,2^, Sander M.J. van Kuijk^3^, Floortje Mols^2,4^, Fränzel J.B. van Duijnhoven^5^, Renate M. Winkels^6^, Huub Hoofs^7^, IJmert Kant^7^, Luc J. Smits^7^, Stéphanie O. Breukink^8^, Lonneke V. van de Poll-Franse^3,4,9^, Ellen Kampman^5^, Sandra Beijer^4^, Matty P. Weijenberg^1^, Martijn J.L. Bours^1^

**Author affiliations**

^1^ Department of Epidemiology, GROW – School for Oncology and Developmental Biology, Maastricht University, P. Debyeplein 1, 6200 MD Maastricht, the Netherlands

^2^ CoRPS – Center of Research on Psychology in Somatic diseases, Department of Medical and Clinical Psychology, Tilburg University, Warandelaan 2, 5037 AB Tilburg, the Netherlands

^3^ Clinical Epidemiology and Medical Technology Assessment, Maastricht University Medical Centre+, P. Debyelaan 25, PO Box 5800, Maastricht 6202 AZ, the Netherlands

^4^ Netherlands Comprehensive Cancer Organisation (IKNL), Godebaldkwartier 419, 3511 DT Utrecht, the Netherlands

^5^ Division of Human Nutrition, Wageningen University & Research, Stippeneng 4, 6708 WE Wageningen, the Netherlands

^6^ Department of Public Health Sciences, Penn State Cancer Institute, 500 University Drive Hershey, PA 17033, USA

^7^ Department of Epidemiology, CAPHRI School for Public Health and Primary Care, Faculty of Health, Medicine and Life Sciences, Maastricht University, P. Debyeplein 1, 6200 MD Maastricht, the Netherlands

^8^ Department of Surgery, Maastricht University Medical Centre, P. Debyelaan 25, 6229 HX Maastricht, the Netherlands

^9^ Department of Psychosocial Oncology and Epidemiology, Netherlands Cancer Institute, Plesmanlaan 121, 1066 CX Amsterdam, the Netherlands

**Corresponding author:**

Dóra Révész, PhD

Department of Epidemiology, GROW – School for Oncology and Developmental Biology, Maastricht University, P. Debyeplein 1, 6200 MD Maastricht, the Netherlands

[Dora.Revesz@maastrichtuniversity.nl](mailto:Dora.Revesz@maastrichtuniversity.nl) / [D.Revesz@uvt.nl](mailto:D.Revesz@uvt.nl)

T: +31 043 388 2903

F: +31 043 388 4128

| **Supplementary Table 3**: Sensitivity analyses of the seven prediction models for health-related quality of life (HRQoL), with only respective baseline HRQoL values, without baseline HRQoL and with the complete models. | | | |
| --- | --- | --- | --- |
| **HRQoL domains** | **AUC only baseline HRQoL** | **AUC without baseline HRQoL** | **AUC complete models** |
| Global quality of life | 0.82 | 0.82 | 0.84 |
| Cognitive functioning | 0.86 | 0.80 | 0.87 |
| Emotional functioning | 0.83 | 0.86 | 0.86 |
| Physical functioning | 0.92 | 0.88 | 0.93 |
| Role functioning | 0.80 | 0.83 | 0.83 |
| Social functioning | 0.81 | 0.85 | 0.86 |
| Fatigue | 0.85 | 0.78 | 0.86 |
| **Footnotes**: AUC = Area under the Receiver Operator Characteristic curve | | | |
